# Long-term outcome of a pragmatic trial of multifaceted intervention (STROKE-CARD care) to reduce cardiovascular risk and improve quality-of-life after ischaemic stroke and transient ischaemic attack: study protocol

**DOI:** 10.1186/s12872-022-02785-5

**Published:** 2022-08-01

**Authors:** Christian Boehme, Lena Domig, Silvia Komarek, Thomas Toell, Lukas Mayer, Benjamin Dejakum, Stefan Krebs, Raimund Pechlaner, Alexandra Bernegger, Christoph Mueller, Gerhard Rumpold, Andrea Griesmacher, Marion Vigl, Gudrun Schoenherr, Christoph Schmidauer, Julia Ferrari, Wilfried Lang, Michael Knoflach, Stefan Kiechl

**Affiliations:** 1 Department of Neurology, Medical University of Innsbruck, Anichstrasse 35, 6020 Innsbruck, Austria; 2 VASCage, Research Centre On Vascular Ageing and Stroke, Innsbruck, Austria; 3 Department of Neurology, Hospital St. John’s of God, Vienna, Austria; 4 Department of Medical Psychology, Medical University of Innsbruck, Innsbruck, Austria; 5 Central Institute of Medical and Chemical Laboratory Diagnostics, University Hospital of Innsbruck, Innsbruck, Austria; 6 Medical Faculty, Sigmund Freud Private University, Vienna, Austria

**Keywords:** Ischaemic stroke, Transient ischaemic attack, Stroke secondary prevention, Disease management program, Stroke long-term follow-up

## Abstract

**Background:**

Patients with ischaemic stroke or transient ischaemic attack (TIA) are at high risk of incident cardiovascular events and recurrent stroke. Despite compelling evidence about the efficacy of secondary prevention, a substantial gap exists between risk factor management in real life and that recommended by international guidelines. We conducted the STROKE-CARD trial (NCT02156778), a multifaceted pragmatic disease management program between 2014 and 2018 with follow-up until 2019. This program successfully reduced cardiovascular risk and improved health-related quality of life and functional outcome in patients with acute ischaemic stroke or TIA within 12 months after the index event. To investigate potential long-term effects of STROKE-CARD care compared to standard care, an extension of follow-up is warranted.

**Methods:**

We aim to include all patients from the STROKE-CARD trial (n = 2149) for long-term follow-up between 2019 and 2021 with the study visit scheduled 3–6 years after the stroke/TIA event. The co-primary endpoint is the composite of major recurrent cardiovascular events (nonfatal stroke, nonfatal myocardial infarction, and vascular death) from hospital discharge until the long-term follow-up visit and health-related quality of life measured with the European Quality of Life-5 Dimensions (EQ-5D-3L) at the final visit. Secondary endpoints include overall mortality, long-term functional outcome, and target-level achievement in risk factor management.

**Discussion:**

This long-term follow-up will provide evidence on whether the pragmatic post-stroke/TIA intervention program STROKE-CARD is capable of preventing recurrent cardiovascular events and improving quality-of-life in the long run.

*Trial registration* clinicaltrials.gov: NCT04205006 on 19 December 2019.

## Background

Stroke is the second leading cause of death worldwide and one of the leading contributors to disability [1, 2]. Furthermore, the life-time risk of stroke in people over 25 years of age is approximately 25%. [3] While the age-specific incidence of stroke is decreasing in most high-income countries, the absolute number of stroke patients is still rising, mainly based on continuous population aging and growth [1]. Stroke survivors and TIA patients are at high risk of suffering from further cardiovascular events and stroke. The risk for a recurrent stroke is high at about 10% within the first year after the stroke event and more than 25% within the first 5 years [4]. Recurrent strokes account for about one quarter of stroke events in nationwide registries [5]. This subgroup tends to have a worse functional outcome and elevated risk of death [6]. Persisting deficits and potentially avoidable long-term post-stroke complications are significant contributors to functional impairment and poor quality of life in the long run after ischaemic stroke and TIA and therefore are an appealing target for concerted interventions.

There is compelling evidence that > 90% of ischaemic strokes are attributable to poor lifestyle and modifiable risk factors and most patients with acute stroke have one or more un- or undertreated risk factors [7, 8] Evidence-based behavioural and pharmacological secondary prevention strategies were estimated to reduce the risk of recurrent cardiovascular events by more than 80% [9]. However, real-world data show that prevention goals and risk factor target levels are rarely achieved [8, 10–15]. Thus, there is a significant gap between evidence-based stroke prevention on the one hand and realization of preventive measures and target level achievements in the real world on the other. The gap even broadens in patients with recurrent stroke or TIA [8]. Also, discontinuation of prescribed medications seems to be an uphill battle in post-stroke care and applies to roughly one-third of stroke patients within the first year after the stroke event [16, 17].

The recently published STROKE-CARD trial, a multifaceted pragmatic post-stroke disease management program reduced rates of recurrent cardiovascular events by one third (HR 0.63; 95% CI 0.45–0.88; *P* = 0.007). The number needed to treat to prevent one major CVD event in the first year after the stroke or TIA was 35 (95% CI 19–154). Also, quality of life in stroke and TIA-patients was improved in the first year after the index event. The median EQ-5D-3L health utility score was 0.783 (IQR 0.687–1.000) in the STROKE-CARD care group and 0.779 (IQR 0.573–1.000) in the standard care group (*P* < 0.001) [18, 19]. To investigate potential long-term effects of STROKE-CARD care an extension of follow-up of the trial patients is warranted.

Acute and short-term management of stroke and TIA has improved tremendously over the past years with substantial advances in acute therapy, implementation of comprehensive pathways for stroke and TIA, and approval of novel highly effective preventive treatments. As a decisive unmet challenge in stroke medicine, strategies of long-term care have to be developed and rigorously tested in order to maintain improved short-term patient outcome in the long run and the STROKE-CARD concept holds promise here.

## Methods

### Study objectives

The main objective is to gain scientific proof that the disease management program STROKE-CARD [18, 19] ameliorates patient wellbeing (health-related quality of life) and reduces recurrent cardiovascular events among stroke or TIA patients over a period of 3–6 years after the index event.

Secondary objectives include long-term functional outcome over longer term after participation in the STROKE-CARD trial, a detailed assessment of patient adherence to drug prescriptions and identification of subgroups with the most pronounced benefit, determination of sustainability of benefits of STROKE-CARD care over longer term and target level achievement of cardiovascular risk factors.

### Study design and centres

The study is designed as a long-term follow-up of the randomised patients included in the STROKE-CARD trial with blinded outcome assessment. STROKE-CARD was a pragmatic block-randomised controlled open-label trial with blinded outcome assessment using standard care versus STROKE-CARD care. The differences in care were described previously [18, 19]. The study was registered with clinicaltrials.gov (NCT04205006) before recruitment on December 19th 2019. The two study centres are the Departments of Neurology at the University Hospital Innsbruck and the Hospital St. John of God in Vienna, Austria. The department in Innsbruck serves as the comprehensive tertiary stroke centre for the entire federal state of Tyrol (catchment area approximately one million inhabitants) and as the primary stroke unit for the city of Innsbruck and 65 surrounding suburban communities with approximately 300,000 inhabitants. Patients from this exclusive catchment area represent an entirely unselected cohort of stroke and TIA patients [5]. The Hospital St. John of God in Vienna serves as one of the three comprehensive stroke units in Vienna, Austria. The Viennese network includes five additional primary stroke units and serves the city of Vienna with a catchment area of approximately 1.9 million inhabitants.

### Study population

The study population comprises all patients with acute ischaemic stroke or TIA (ABCD2-Score ≥ 3) who took part in the STROKE-CARD trial [18, 19]. In brief, the exclusion criteria for the STROKE-CARD trial were patients living outside the survey area and those with permanent severe disability (modified Rankin Scale [mRS] = 5), life expectancy < 1 year, drug addiction or alcohol abuse [20]. We aim to contact every patient (n = 2149) for recruitment, except for those who aborted or deceased within the previous STROKE-CARD trial. Patients will be informed about goals, procedures and potential risks of the study. They will be invited to a single out-patient visit at our departments. If the patient is unable or unwilling to travel, the study visit will be done by phone. We will gather medical reports from general practitioners and the Tyrol’s hospitals. All patients included in the follow-up study will provide written informed consent. In case we fail to trace a patient, we will retrieve medical information from his/her general practitioner as well as hospital records. Inclusion and exclusion criteria are listed in Table 1.

**Table 1:** STROKE-CARD long-term follow-up inclusion and exclusion criteria

Inclusion criteria	Exclusion criteria
Inclusion in the previous STROKE-CARD trial Written informed consent	None

### STROKE-CARD intervention

Standard care involved in-hospital patient counselling and education, dietary advice, smoking cessation support, printed information materials and a detailed discharge-from-hospital report (including patient-tailored target levels for risk factor management) to the general practitioner and the patient. Patients with persisting deficits were transferred to rehabilitation services within the scope of the local stroke pathways [5]. Selected high-risk patients (e.g. patients after carotid surgery) were seen in the outpatient clinics. As part of the Austrian Stroke Unit Registry study nurses conducted routine telephone interviews to assess the patients’ 3-month functional outcome [21].

The intervention from the previous STROKE-CARD trial was published previously [18, 19]. In short, additionally to standard care, STROKE-CARD care involved an outpatient appointment for patients and care-givers scheduled three months after the index event and lasting two to three hours. This additional appointment was performed by a multidisciplinary team of stroke physicians, nurses, physiotherapists, occupational and speech therapists. It had the following aims: (a) to reevaluate stroke/TIA aetiology (with potential changes in prevention strategies), (b) to reassess risk factor levels and optimize secondary prevention (adapting medication lists and reinforcing drug adherence), (c) to systematically screen for post-stroke complications and other health problems (holistic approach with initiation of therapy and/or referral to specialists), (d) to assess rehabilitation demands (with referral to rehabilitation services), (e) to manage new-onset cardiovascular disease (CVD) and warning signs of imminent CVD (including referral to revascularization procedures if appropriate), and (f) to enhance patient self-empowerment and knowledge  about CVD and to counsel patients on all matters raised by themselves or their care-givers. Patients and care-givers were also given access to the web-based patient portal “MyStrokecard” for risk factor monitoring, ascertainment of post-stroke complications, and extended patient education. They also were offered to contact the study personnel in case of health problems. They received training for this e-tool during hospital stay with a tailored composition according to individual risk profiles and target levels and introduction to easily applicable screening tools for post-stroke complications.

### Study procedures

Informed consent will be gathered by a consultant neurologist within the study team staff. All other study procedures will be undertaken by the members of the study team staff, who are trained for the specific tasks (consultant neurologists, resident neurologists, PhD students, nurses). All patients from each arm of the STROKE-CARD trial will receive the same procedures in the follow-up study.

We will assess multiple items using different questionnaires: health-related quality of life using the European Quality of Life-5 Dimensions EQ-5D-3L overall health utility score [22, 23]; mood and anxiety will be assessed using the Beck Depression Inventory (BDI) and the Hospital Anxiety and Depression Scale (HADS) [24, 25]; fatigue will be assessed using the Fatigue Severity Scale [26] and incontinence via the Overactive Bladder Symptom Score (OABSS) [27]. Additionally, activities and sports will be assessed using the Baecke Score [28], falls with a fall diary and patient satisfaction using the Post-Stroke checklist [29]. Activities of daily living will be assessed using the Barthel index [30]. In case of seizures we will use the Seizure Severity Questionnaire for assessment [31]. In case of active smoking, a Fagerstroem-test will be handed out to the patient [32]. Cognitive disorders will be assessed using the Mini Mental State Examination (MMSE) [33], the Montreal Cognitive Assessment (MoCA) [34] and the DSM-V-criteria for cognitive disorders [35].

The neurological assessment includes the National Institutes of Health Stroke Scale (NIHSS) [36] and modified Rankin scale (mRS) [20] to screen for residual deficits and patient outcome. We will perform an assessment of social status including nursing allowance and medical history since the last study visit of the STROKE-CARD trial, including reports from other hospitals, general practitioners and specialists will be used for assessment of vascular outcomes and other incident diseases e.g. myocardial infarction, angina, strokes (ischaemic and haemorrhagic) transient ischaemic attacks, revascularization procedures and vascular interventions, new comorbidities, hospital admissions and history of seizures, syncopes, falls and fractures. Additionally, a structured headache history will be assessed. The SPIRIT flow diagram of study procedures is depicted in Table 2.

**Table 2:** SPIRIT flow diagram of study procedures

Timepoint	Allocation in former trial	Enrolment and post-allocation	
	2014–2018	Before visit	Follow-up visit
Enrolment			
Eligibility screen		X	
Informed consent			X
Allocation	X		
Interventions			
Laboratory exam incl. biobank			X
Functional and neurological status			X
Sonography exam			X
Cognition exam			X
Questionnaires			X
Heart rhythm analysis			X
Assessments			
Demographic data			X
Composite CVD endpoint			X
Health-related QoL			
Vascular events			
All-cause mortality			
Target levels of risk factors			
Functional outcome			

*CVD* cardiovascular disease, *QoL* quality of life

The allocation to standard care and STROKE-CARD care was done in the former STROKE-CARD trial. In this follow-up study, all patients will receive the same procedures

We will assess cardiovascular risk factors and the respective target regarding blood pressure measurements, biometric measures (weight, height, body-mass index, waist-to-hip ratio), smoking and alcohol intake (cigarettes per day and pack-years as well as grams of alcohol per day). Laboratory examinations include blood sugar levels, glycated haemoglobin, cholesterol parameters, kidney function, electrolytes, inflammation and coagulation parameters as well as liver enzymes and a full blood cell count. Additionally, the Framingham Risk Score will be assessed at the follow-up visit [37]. Target levels of cardiovascular risk factors are depicted in Table 3.

**Table 3:** Conditions and target levels of cardiovascular risk factors

Condition	Target
Hypertension	BP < 140/90 mmHg
	BP < 130/85 mmHg in patients with diabetes, renal impairment or small-vessel disease [38]
Dyslipidaemia	LDL-C < 70 mg/dL [39–41]
Diabetes	HbA_1c_ < 7% (less stringent target HbA_1c_ in selected individuals^b^) [42]
Smoking	Nicotine abstinence [43]
Physical inactivity	Physical activity of moderate to vigorous intensity with an average of 40 min at least three times per week [43]
Non-adherence to drug prescriptions	Adherence to drug prescription (proportion of days covered ≥ 90%)
Indication for oral anticoagulation	INR 2–3 for atrial fibrillation, INR 2–3 for mechanical aortic valves, INR 2.5–3.5 for mechanical mitral valves; regular use and accurate dose of novel oral anticoagulants

*BP* blood pressure, *LDL-C* low density lipoprotein cholesterol, *HbA*_*1c*_ glycated haemoglobin

^a^Patients with intra- or extracranial vessel stenosis, instable plaques, atherothrombotic strokes, atherosclerotic cardiovascular comorbidities or Diabetes

^b^HbA_1c_-level < 7.0% for most non-pregnant adults or < 6.5% in selected individual patients if this can be achieved without significant hypoglycaemia or other adverse effects of treatment (i.e., polypharmacy) or < 8% in patients with a history of severe hypoglycaemia, limited life expectancy, advanced microvascular or macrovascular complications, extensive comorbid conditions, or long-standing diabetes in whom the goal is difficult to achieve [42]

We will assess cervical artery atherosclerosis, plaque burden and possible stenosis by high resolution ultrasound. Heart rhythm analysis will be performed using the Fibricheck® software (a registered medical device IIa, certified under Directive 93/42/EEC and all software is compliant to ISO62304) via a tablet computer [44]. In case of abnormalities, an electrocardiogram (ECG) will be performed for further diagnosis.

Blood samples are drawn after an overnight fast and at least 12-h abstinence from smoking and are immediately processed and used for routine testing and establishment of a biobank (plasma, serum, whole blood). The samples will be archived in safeguarded freezers at − 80 °C. The temperature is permanently monitored by the hospital’s technical service unit with backup capacity available in case of a technical defect. Sample storage complies with the OECD guidelines [45] and is in accordance with the national Bioethics Commission Report (recommendations of the Austrian bioethics commission) [46].

## Outcomes

### Primary outcomes (co-primary endpoint)

- Composite CVD defined as nonfatal ischaemic stroke, nonfatal haemorrhagic stroke, nonfatal myocardial infarction, or vascular death between hospital discharge and the long-term follow-up visit.
- Self-reported health-related quality of life at the long-term follow-up visit quantified with the European Quality of Life 5-Dimensions 3-Levels (EQ-5D-3L) overall health utility score with rescaled European visual analogue scale weights.

The composite CVD outcomes will be reviewed and assessed by an outcome adjudication committee blinded to patient allocation in the former STROKE-CARD trial.

### Secondary outcomes

- Composite CVD outcome of ischaemic stroke, haemorrhagic stroke, or transient ischaemic attack (TIA) (defined as transient neurological deficit < 24 h and absence of DWI [diffusion-weighted imaging] positive lesions in MRI)
- All-cause mortality
- Individual 3-level components of the European Quality of Life 5-Dimensions 3-Levels (EQ-5D-3L) questionnaire (i.e. mobility, self-care, usual activities, no pain or discomfort, no anxiety or depression) comparing people reporting no problems (level 1) with those reporting problems (level 2 and 3) at the study visit [22, 23].
- Proportions of participants achieving target levels of risk factors in each trial arm of the previous STROKE-CARD trial, including
  - achieving a systolic blood pressure < 140 mmHg and a diastolic blood pressure < 90 mmHg at the 12-month visit or a systolic blood pressure < 130 mmHg and a diastolic blood pressure < 85 mmHg at the study visit in patients with diabetes mellitus, severe renal impairment (defined as estimated glomerular filtration rate < 30 mL/min/1.73 m^2^), or small-vessel disease at baseline [38],
  - Achieving a glycated haemoglobin (HbA_1c_) concentration < 7.0% at the study visit in patients with diabetes mellitus, or less stringent targets in selected individuals [42]
  - Having quit smoking by the study visit in patients that had been smokers at baseline in the STROKE-CARD trial [43],
  - Being physically active for an average of 40 min at least three times per week assessed with the Baecke questionnaire based on the questions on the number of hours of sports activities per week, months doing this sport in a year, minutes spent walking during leisure time, and minutes spent cycling during leisure time [28],
  - Taking lipid-lowering medication in all patients except those with an ischaemic stroke or TIA of other determined aetiology (e.g. index event due to vasculitis or carotid artery, vertebral artery, or aortic dissection) [43],
  - Achieving a low-density lipoprotein (LDL) cholesterol concentration < 70 mg [39–41],
  - Taking anticoagulation or antiplatelet therapy in patients that had been prescribed such medication [43],
  - Taking anticoagulation in patients with atrial fibrillation or mechanical heart valves [43];
  - Good functional outcome defined as modified Rankin Scale (mRS) ≤ 2,
  - Distribution across mRS categories at the study visit (“shift analysis”).

Functional outcome on the mRS will be assessed by study team members blinded to allocation in the former STROKE-CARD trial.

### Statistical analyses

This study will assess the efficacy of STROKE-CARD care in preventing recurrent major cardiovascular events and improving health-related quality of life over a follow-up period of 3–6 years. All patients that participated in STROKE-CARD (n = 2.149) will be invited for participation in the current trial. Power calculations underlying STROKE-CARD have been published previously [18, 19]. We aim to gather information on all former trial patients (= 2.149). For an expected mean time of follow-up of four yours and equal event rates, the power (α = 0.05) will be 99% to detect effect of the 1-year analysis (HR = 0.63) and 82% for a HR = 0.74.

The same co-primary efficacy end-points as in STROKE-CARD will be used, comprising incidence of major recurrent cardiovascular events (composite of nonfatal ischaemic stroke, nonfatal myocardial infarction, or vascular death) and health-related quality of life as measured by the EQ-5D-3L health utility score [23]. Effects of STROKE-CARD care on the risk of major recurrent cardiovascular events will be estimated using Cox regression stratified for trial centres. The proportional hazards assumption will be tested using Schoenfeld residuals. Differences in EQ-5D-3L between STROKE-CARD care and standard care will be tested by Mann Whitney U-test. We will consider STROKE-CARD care as effective over an extended time period if the analysis of both co-primary endpoints yields two-sided *P* values ≤ 0.05. The analysis is unadjusted and will be stratified by trial centre. In a sensitivity analysis, effect sizes will be adjusted for age at hospital discharge, sex, type of index event (stroke vs. transient ischaemic attack), in addition to trial centre (Innsbruck, Vienna). Further adjusted analyses will be reported in case the two trial arms differ according to any other baseline characteristics. Effects of STROKE-CARD care on dichotomous outcomes such as EQ-5D-3L subcategories will be performed by calculating risk ratios by means of Poisson regression with robust error variance adjusted for trial centre, and effects on functional outcome as assessed by modified Rankin Scale score by means of ordinal logistic regression. Pre-specified sensitivity analyses include modelling of CVD outcomes with multivariable adjustment for age, sex, and type of index event, as-treated analysis, and sub-group analyses defined by sex, age (< 70 vs. ≥ 70 years), type of index event, use of the “MyStrokecard” web application, study centre, and after exclusion of patients whose index event was a TIA with an ABCD2 score of 3 points. For analyses of secondary endpoints and subgroups, the Benjamini–Hochberg procedure will be used to adjust for multiple testing.

## Discussion

The risk of future cardiovascular events and poor quality of life is high after ischaemic stroke and TIA. The STROKE-CARD trial is the first large-scale randomized trial which reduced recurrent cardiovascular events (by approximately one third [5.4% vs. 8.3%]) and improved quality of life (*p* < 0.001) within 1 year after ischaemic stroke or TIA [19]. The intervention is pragmatic and thus easily to implement in clinical routine. It pursues the concept that optimal acute stroke care by the multidisciplinary stroke team does not stop when patients are discharged from hospital but extends to a thorough three-month assessment and individualized counselling. The intervention comprised an outpatient appointment for patients and care-givers scheduled three months after the index event to reevaluate stroke/TIA, to reassess risk factor levels and optimize secondary, to systematically screen for post-stroke complications and other health problems and to enhance patient self-empowerment and knowledge about CVD and to counsel patients on all matters raised by themselves or their care-givers.

Our previous trial also showed benefits in functional outcome 1 year after the index event. We now strive for an extension of follow-up up to 36–72 months for each participant and to prove whether the trial’s success is maintained in the long run.

### Other long-term post-stroke disease management programs

Previous trials on long-term secondary prevention strategies and disease management programs in ischaemic stroke or TIA patients have shown variable improvements in risk factor control but most of them did not analyse potential effects on recurrent cardiovascular disease and stroke events. An overview on completed and ongoing trials focusing on multimodal secondary stroke prevention and disease management is provided in Table 4.

**Table 4:** Previous trials using post-stroke disease management programs and strategies with follow-up ≥ 2 years

Trial, country	Year	Inclusion criteria	n	Age (y)	Intervention type/model	FU (mo)	Outcome Measures	Significant results
CEOPS, France [47]	2021	Ischaemic or haemorrhagic stroke, TIA, > 40 y	Target = 410	N/A	Education & self-management, nurse-led, telephone-based, visits at 6, 12 and 24 mo	24	BP, RF-control, QoL, cognitive function	Ongoing
INSPiRE-TMS, Germany [48]	2019	TIA and minor stroke (mRS ≤ 2), age > 18 y	2.098	67	RF-management & support program, up to 8 visits	24	Stroke, ACS, RF-control, mortality, hospitalisations	Improved achievement of target levels
STANDFIRM, Australia [49]	2017	Ischaemic or haemorrhagic stroke, TIA, > 18 y	563	70	Community-based intervention, evidence-based care plan, educational sessions, telephone assessments	24	Targets for cardiometabolic factors	Improved Cholesterol levels
Kono et al., Japan [50]	2013	Ischaemic stroke, (mRS 0–1), non-cardioembolic origin	70	64	Lifestyle intervention program with counselling at BL, 3, 6 mo, exercise training for 24 weeks	36	Vascular death, hospitalization for vascular cause, RF-control	Vascular events, physical activity, BP-lowering, salt-intake
Fukuoka et al., Japan [51]	2019	Ischaemic stroke (mRS < 4) within last year, TIA, age 40–80 y	321	67	Education & self-management, individualized target values, therapeutic activities, 10 telephone assessments	30	FRS, Stroke recurrence, CVD, all-cause mortality, vascular events	none
Hedman et al., Sweden [52]	2019	Ischaemic stroke	145	67	Client-centred ADL care	60	Independence in ADL, life satisfaction	none
STROKE-CARD Long-term follow-up, Austria	2021	Acute ischaemic stroke (mRS 0–4) or TIA (ABCD^2^ ≥ 3); > 18 y	Target = 2.149	72	3 mo clinical visit with RF-assessment, online RF-monitoring, 12 mo visit	36–72	Cardiovascular events, vascular death, QoL, RF-control	Ongoing

Ongoing and completed trials in post-stroke disease management. The year indicates either the completion date or the estimated completion date. Age indicates mean or median age of the study population

*TIA* transient ischaemic attack, *y* years, *mRS* modified Rankin scale, *n* number, *N/A* not available, *mo* months, *RF* risk factor, *BL* baseline, *ADL* activities of daily living, *FU* follow-up, *BP* blood pressure, *QoL* quality of life, *ACS* acute coronary syndrome, *FRS* Framingham risk score, *CVD* cardiovascular disease

The largest long-term follow up study for ischaemic stroke and TIA patients to date, the INSPiRE-TMS trial, showed various improvements in target levels of cardiovascular risk factors and improvement in functional outcome in subgroups after a follow up of 2 years but did not show any difference in recurrent vascular events. The study focused on minor stroke and TIA patients and moderate or severe strokes were excluded [48]. A recent Australian study reported improved cholesterol levels in ischaemic or haemorrhagic stroke patients after 2 years but was not designed and powered to detect differences in recurrent vascular events [49]. A trial from Japan had a different strategy and focused on lifestyle intervention and exercise training. The program reduced recurrent vascular events and improved risk factor target levels but had a limited sample size. However, the intervention that lasted for six months showed benefits after a 3 year follow-up [50]. Other studies showed no significant effects in risk factor target levels and were not designed or powered to detect differences in incident cardiovascular disease events [51, 52].

The intervention models in these post-stroke disease management programs varied widely concerning intensity and type of measures e.g. community-based interventions, nurse-led and educational interventions at various timepoints. Our STROKE-CARD concept is a multidisciplinary but lean and cheap intervention leveraging contemporary e-technology and encouraging patient self-empowerment with the prospect of a widespread implementation.

### Strengths and limitations

The unique features of the post-stroke disease management program STROKE-CARD rely on the comprehensive focus on both recurrent vascular events and post-stroke complications as well as health-related quality of life after stroke. Furthermore, the study included patients with various disease severity ranging from moderate risk TIA to severe strokes.

Our program was developed in a country with universal health care access, and all but two patients in the STROKE-CARD trial were of European descent [8]. Therefore, potential findings may not necessarily apply to ethnically more diverse populations or populations with limited health care access and are probably not easy to extrapolate to less developed stroke care systems. Nevertheless, the benefit of a lean and easy implementable post-stroke disease management program like STROKE-CARD might be even higher in low-quality stroke care systems.

The STROKE-CARD concept is currently implemented as a standard of care in parts of Austria with the prospect of implementation in other European countries and possibly beyond.

### Trial status

The study protocol version 2.0 from October 25th 2019 was approved by the ethics committee of the Medical University of Innsbruck on November 11th 2019. Recruitment was initiated on December 19th 2019. Recruitment completion is expected for May 2022.

## Data Availability

Data sharing is not applicable to this article as no datasets were generated or analysed during the current study.

## Abbreviations

BP: Blood pressure
CVD: Cardiovascular disease
DWI: Diffusion-weighted imaging
HbA_1c_: Glycated haemoglobin
HDL: High density lipoprotein
LDL: Low density lipoprotein
MRI: Magnetic resonance imaging
mRS: Modified Rankin Scale
NIHSS: National Institutes of Health Stroke Scale
RR: Risk ratios
TIA: Transient ischaemic attack

## References

[1] GBD 2016 Stroke Collaborators (2019). Global, regional, and national burden of stroke, 1990–2016: a systematic analysis for the Global Burden of Disease Study 2016. Lancet Neurol.

[2] GBD 2015 DALYs and HALE Collaborators (2016). Global, regional, and national disability-adjusted life-years (DALYs) for 315 diseases and injuries and healthy life expectancy (HALE), 1990–2015: a systematic analysis for the Global Burden of Disease Study 2015. Lancet.

[3] Feigin VL, Nguyen G, Cercy K, Johnson CO, Alam T, GBD 2016 Lifetime Risk of Stroke Collaborators (2018). Global, regional, and country-specific lifetime risks of stroke, 1990 and 2016. N Engl J Med.

[4] Mohan KM, Wolfe CDA, Rudd AG, Heuschmann PU, Kolominsky-Rabas PL, Grieve AP (2011). Risk and cumulative risk of stroke recurrence: a systematic review and meta-analysis. Stroke.

[5] Willeit J, Geley T, Schöch J, Rinner H, Tür A, Kreuzer H (2015). Thrombolysis and clinical outcome in patients with stroke after implementation of the Tyrol Stroke Pathway: a retrospective observational study. Lancet Neurol.

[6] Samsa GP, Bian J, Lipscomb J, Matchar DB (1999). Epidemiology of recurrent cerebral infarction: a medicare claims-based comparison of first and recurrent strokes on 2-year survival and cost. Stroke.

[7] Feigin VL, Roth GA, Naghavi M, Parmar P, Krishnamurthi R, Chugh S (2016). Global burden of stroke and risk factors in 188 countries, during 1990–2013: a systematic analysis for the Global Burden of Disease Study 2013. Lancet Neurol.

[8] Boehme C, Toell T, Mayer L, Domig L, Pechlaner R, Willeit K (2019). The dimension of preventable stroke in a large representative patient cohort. Neurology.

[9] Hackam DG, Spence JD (2007). Combining multiple approaches for the secondary prevention of vascular events after stroke: a quantitative modeling study. Stroke.

[10] Heuschmann PU, Kircher J, Nowe T, Dittrich R, Reiner Z, Cifkova R (2015). Control of main risk factors after ischaemic stroke across Europe: data from the stroke-specific module of the EUROASPIRE III survey. Eur J Prev Cardiol.

[11] Brewer L, Mellon L, Hall P, Dolan E, Horgan F, Shelley E (2015). Secondary prevention after ischaemic stroke: the ASPIRE-S study. BMC Neurol.

[12] Gladstone DJ, Bui E, Fang J, Laupacis A, Lindsay MP, Tu JV (2009). Potentially preventable strokes in high-risk patients with atrial fibrillation who are not adequately anticoagulated. Stroke.

[13] George MG, Tong X, Bowman BA (2017). Prevalence of cardiovascular risk factors and strokes in younger adults. JAMA Neurol.

[14] Morren JA, Salgado ED (2012). Prevalence and control of stroke risk factors in a South Florida population. Int J Neurosci.

[15] Fisher M, Moores L, Alsharif MN, Paganini-Hill A (2016). Definition and implications of the preventable stroke. JAMA Neurol.

[16] Bushnell CD, Olson DM, Zhao X, Pan W, Zimmer LO, Goldstein LB (2017). Secondary preventive medication persistence and adherence 1 year after stroke. Neurology.

[17] Dalli LL, Kim J, Thrift AG, Andrew NE, Sanfilippo FM, Lopez D (2021). Patterns of use and discontinuation of secondary prevention medications after stroke. Neurology.

[18] Toell T, Boehme C, Mayer L, Krebs S, Lang C, Willeit K (2018). Pragmatic trial of multifaceted intervention (STROKE-CARD care) to reduce cardiovascular risk and improve quality-of-life after ischaemic stroke and transient ischaemic attack -study protocol. BMC Neurol.

[19] Willeit P, Toell T, Boehme C, Krebs S, Mayer L, Lang C (2020). STROKE-CARD care to prevent cardiovascular events and improve quality of life after acute ischaemic stroke or TIA: a randomised clinical trial. EClinicalMedicine.

[20] Van Swieten JC, Koudstaal PJ, Visser MC, Schouten HJ, Van Gijn J (1988). Interobserver agreement for the assessment of handicap in stroke patients. Stroke.

[21] Mayer L, Ferrari J, Krebs S, Boehme C, Toell T, Matosevic B (2018). ABCD3-I score and the risk of early or 3-month stroke recurrence in tissue- and time-based definitions of TIA and minor stroke. J Neurol.

[22] Greiner W, Weijnen T, Nieuwenhuizen M, Oppe S, Badia X, Busschbach J (2013). A single European currency for EQ-5D health states. Eur J Health Econ.

[23] Szende A, Janssen B, Cabases J (2014). Self-reported population health: an international perspective based on EQ-5D.

[24] Beck AT (1961). An inventory for measuring depression. Arch Gen Psychiatry.

[25] Zigmond AS, Snaith RP (1983). The hospital anxiety and depression scale. Acta Psychiatr Neurol Scand.

[26] Krupp LB (1989). The fatigue severity scale: application to patients with multiple sclerosis and systemic lupus erythematosus. Arch Neurol.

[27] Homma Y, Yoshida M, Seki N, Yokoyama O, Kakizaki H, Gotoh M (2006). Symptom assessment tool for overactive bladder syndrome-overactive bladder symptom score. Urology.

[28] Baecke JA, Burema J, Frijters JE (1982). A short questionnaire for the measurement of habitual physical activity in epidemiological studies. Am J Clin Nutr.

[29] Ward AB, Chen C, Norrving B, Gillard P, Walker MF, Blackburn S (2014). Evaluation of the post stroke checklist: a pilot study in the United Kingdom and Singapore. Int J Stroke.

[30] Collin C, Wade DT, Davies S, Horne V (1988). The Barthel ADL index: a reliability study. Int Disabil Stud.

[31] Cramer JA. Seizure Severity Questionnaire V2.2. 2010 https://www.epilepsy.com/sites/core/files/atoms/files/SSQ%20BL%2BFU%20for%20Academic%20use.pdf. Accessed 10 Feb 2021.

[32] Heatherton TF, Kozlowski LT, Frecker RC, Fagerstrom KO (1991). The Fagerstrom test for nicotine dependence: a revision of the Fagerstrom tolerance questionnaire. Addiction.

[33] Folstein MF, Folstein SE, McHugh PR (1975). Mini-mental state. J Psychiatr Res.

[34] Nasreddine ZS, Phillips NA, Bédirian V, Charbonneau S, Whitehead V, Collin I (2005). The Montreal Cognitive Assessment, MoCA: a brief screening tool for mild cognitive impairment. J Am Geriatr Soc.

[35] American Psychiatric Association (2013). Diagnostic and statistical manual of mental disorders, DSM-5TM.

[36] Lyden P, Brott T, Tilley B, Welch KM, Mascha EJ, Levine S (1994). Improved reliability of the NIH Stroke Scale using video training. NINDS TPA Stroke Study. Group Stroke.

[37] Wilson PWF, D’Agostino RB, Levy D, Belanger AM, Silbershatz H, Kannel WB (1998). Prediction of coronary heart disease using risk factor categories. Circulation.

[38] ESH/ESC Guidelines for the management of arterial hypertension (2013). The Task Force for the management of arterial hypertension of the European Society of Hypertension (ESH) and of the European Society of Cardiology (ESC). Eur Heart J.

[39] Grundy SM, Stone NJ, Bailey AL, Beam C, Birtcher KK, Blumenthal RS (2019). 2018 AHA/ACC/AACVPR/AAPA/ABC/ACPM/ADA/AGS/APhA/ASPC/NLA/PCNA guideline on the management of blood cholesterol: executive summary: a report of the American College of Cardiology/American Heart Association Task Force on clinical practice guidelines. J Am Coll Cardiol.

[40] Grundy SM, Stone NJ, Bailey AL, Beam C, Birtcher KK, Blumenthal RS (2019). 2018 AHA/ACC/AACVPR/AAPA/ABC/ACPM/ADA/AGS/APhA/ASPC/NLA/PCNA guideline on the management of blood cholesterol: a report of the American College of Cardiology/American Heart Association Task Force on clinical practice guidelines. Circulation.

[41] Toplak H, Ludvik B, Lechleitner M, Dieplinger H, Föger B, Paulweber B (2016). Austrian Lipid Consensus on the management of metabolic lipid disorders to prevent vascular complications: a joint position statement issued by eight medical societies. 2016 update. Wien Klin Wochenschr.

[42] American Diabetes Association (2019). 6. Glycemic targets: standards of medical care in diabetes-2019. Diabetes Care.

[43] Kernan WN, Ovbiagele B, Black HR, Bravata DM, Chimowitz MI, Ezekowitz MD (2014). Guidelines for the prevention of stroke in patients with stroke and transient ischemic attack: a guideline for healthcare professionals from the American Heart Association/American Stroke Association. Stroke.

[44] Proesmans T, Mortelmans C, Van Haelst R, Verbrugge F, Vandervoort P, Vaes B (2019). Mobile phone-based use of the photoplethysmography technique to detect atrial fibrillation in primary care: diagnostic accuracy study of the FibriCheck App. JMIR MHealth UHealth.

[45] OECD Organization for Economic Cooperation and Development (2010). OECD guidelines on human biobanks and genetic research databases. Eur J Health Law.

[46] Austrian Bioethics Commission. Biobanks for Medical Research. 2007. https://www.bundeskanzleramt.gv.at/dam/jcr:01702c56-68a2-4f17-a265-dc16ec28caac/Erg%C3%A4nzungen_des_Berichts_der_Bioethikkommission_zu_Biobanekn_d%C3%BCr_die_wissenschaftliche_Forschung_vom_14._M%C3%A4rz_2011.pdf. Accessed 10 Feb 2021.

[47] Mendyk AM, Duhamel A, Bejot Y, Leys D, Derex L, Dereeper O (2018). Controlled Education of patients after Stroke (CEOPS)- nurse-led multimodal and long-term interventional program involving a patient’s caregiver to optimize secondary prevention of stroke: study protocol for a randomized controlled trial. Trials.

[48] Ahmadi M, Laumeier I, Ihl T, Steinicke M, Ferse C, Endres M (2020). A support programme for secondary prevention in patients with transient ischaemic attack and minor stroke (INSPiRE-TMS): an open-label, randomised controlled trial. Lancet Neurol.

[49] Olaiya MT, Kim J, Nelson MR, Srikanth VK, Bladin CF, Gerraty RP (2017). Effectiveness of a shared team approach between nurses and doctors for improved risk factor management in survivors of stroke: a cluster randomized controlled trial. Eur J Neurol.

[50] Kono Y, Yamada S, Yamaguchi J, Hagiwara Y, Iritani N, Ishida S (2013). Secondary prevention of new vascular events with lifestyle intervention in patients with noncardioembolic mild ischemic stroke: a single-center randomized controlled trial. Cerebrovasc Dis Basel Switz.

[51] Fukuoka Y, Hosomi N, Hyakuta T, Omori T, Ito Y, Uemura J (2019). Effects of a disease management program for preventing recurrent ischemic stroke. Stroke.

[52] Hedman A, Eriksson G, von Koch L, Guidetti S (2019). Five-year follow-up of a cluster-randomized controlled trial of a client-centred activities of daily living intervention for people with stroke. Clin Rehabil.

